# T_2_-dependent errors in MOLLI T_1_ values: simulations, phantoms, and in-vivo studies

**DOI:** 10.1186/1532-429X-14-S1-P281

**Published:** 2012-02-01

**Authors:** Kelvin Chow, Jacqueline Flewitt, Joseph J Pagano, Jordin D Green, Matthias G Friedrich, Richard B Thompson

**Affiliations:** 1 Department of Biomedical Engineering, University of Alberta, Edmonton, AB, Canada; 2 Stephenson CMR Centre, University of Calgary, Calgary, AB, Canada; 3 Siemens Healthcare, Calgary, AB, Canada

## Background

Diffuse myocardial fibrosis occurs in various cardiomyopathies and can be indirectly assessed with blood and myocardial T_1_ mapping at baseline and after gadolinium administration. The widely used MOdified Look-Locker Inversion-recovery (MOLLI) [1] sequence is known to underestimate myocardial T_1_ at higher heart rates, but its dependence on T_2_ has not been explored. We investigate MOLLI’s T_1_ accuracy in phantoms and confirm with simulations and in-vivo studies. T_1_ values are further compared with a saturation-recovery T_1_ mapping sequence [2].

## Methods

### Phantoms

14 NiCl_2_ agarose phantoms with a broad range of T_1_ and T_2_ values were imaged with a gold-standard inversion-recovery spin-echo (IR-SE) sequence, MOLLI, and a new SAturation-recovery single-SHot Acquisition (SASHA) technique (Siemens Avanto 1.5T). IR-SE: 16 TIs 100-5000ms, TE=11ms, TR>5s, 90° flip. MOLLI: 2 inversion sets of 3 and 5 images, 75% partial Fourier, TImin=110ms with 80ms increment, 35° flip, TE/TR=1.3/2.9ms, simulated HR=60bpm. SASHA: single-shot SSFP images from 10 consecutive heartbeats with incremented TI spanning the RR interval in the last 9 images (no saturation in the first image), 70° flip, TE/TR=1.3/2.6ms, full k-space, simulated HR=60bpm. T_2_: spin-echo (SE) with 7 TEs 11-200ms. Simulations: Bloch equation simulations of MOLLI and SASHA were performed in MATLAB using actual acquisition and physiology parameters and SE measured T_1_ and T_2_ values.

### In-Vivo

For 10 healthy volunteers (5 male, 28.8±6.6yrs), blood and myocardial T_1_s were measured using MOLLI and SASHA (parameters as above) in a mid-ventricular short-axis slice at baseline and 20 minutes following 0.1mmol/kg Magnevist.

## Results

**Figure 1:**
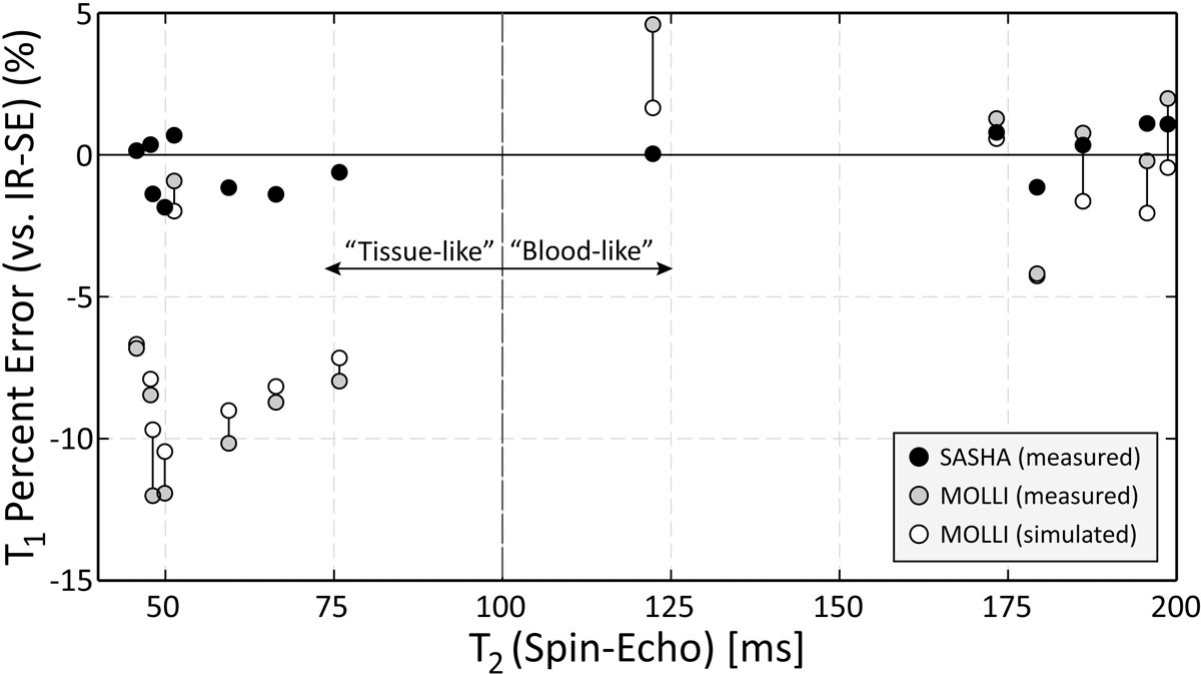
Error in MOLLI and SASHA T_1_ values compared to gold standard inversion-recovery spin-echo (IR-SE) in "tissue-like" phantoms (T_1_s 339-1145ms) and "blood-like" phantoms (T_1_s 275-1452ms). A Bloch equation simulation of MOLLI using actual acquisition and physiology timing parameters is also shown, with the difference between simulated and actual results indicated with a vertical line for each phantom.

In blood-like phantoms with long T_2_ (179-196ms), SASHA and MOLLI T_1_s agree well with IR-SE (0.7±0.5% and 2.2±1.8% absolute difference respectively), while shorter T_2_ (46-76ms) tissue-like phantoms have greater underestimation with MOLLI (8.4±3.5%) than SASHA (0.9±0.6%) (Fig. 1). MOLLI simulations predict underestimated T_1_s, with 1.3±0.9% absolute difference from observed values (vertical lines, Fig. 1). SASHA simulations also agree well with observations (0.8±0.5%, not shown). In healthy volunteers (63.3±8.4bpm), MOLLI T_1_s also show greater underestimation compared to SASHA in tissue than blood, although the difference is larger than observed in phantoms or predicted by simulations in all cases (Table 1).

**Table 1:** Comparison of MOLLI and SASHA T_1_ values in 10 healthy volunteers prior to and 20 minutes following 0.1 mmol/kg Magnevist. All comparisons between MOLLI and SASHA are significant (p<0.01, two-tailed, paired Student’s t-test).

T_1_ [ms]	Myocardium (mean±std)		Blood (mean±std)	
	
	Baseline	Post Gd (20 min)	Baseline	Post Gd (20 min)
**MOLLI**	935.5±24.9	614.4±33.8	1514.1±107.5	524.9±55.2
**SASHA**	1175.2±27.6	752.9±48.2	1687.4±85.8	542.6±56.3

## Conclusions

MOLLI significantly underestimates T_1_s in shorter T_2_ tissue-like phantoms but less so in longer T_2_ blood-like phantoms, as predicted by simulations. Similar trends were observed in-vivo with MOLLI, although with greater T_1_ underestimation (compared to SASHA) than predicted. SASHA had good agreement with IR-SE T_1_ phantom measurements and simulations and can be acquired in less time than MOLLI.

## Funding

N/A
